# The repurposed use of anesthesia machines to ventilate critically ill patients with coronavirus disease 2019 (COVID-19)

**DOI:** 10.1186/s12871-021-01376-9

**Published:** 2021-05-20

**Authors:** Maurizio Bottiroli, Angelo Calini, Riccardo Pinciroli, Ariel Mueller, Antonio Siragusa, Carlo Anelli, Richard D. Urman, Ala Nozari, Lorenzo Berra, Michele Mondino, Roberto Fumagalli

**Affiliations:** 1 Department of Anesthesia and Critical Care, ASST Grande Ospedale Metropolitano Niguarda, P.zza Ospedale Maggiore, 3-, 20162 Milan, Italy; 2 Department of Anesthesia, Critical Care and Pain Medicine, Massachusetts General Hospital, Harvard Medical School, Boston, MA USA; 3 Department of Medicine and Surgery, University of Milan-Bicocca, Milan, Italy; 4 Department of Anesthesiology, Perioperative and Pain Medicine, Brigham and Women’s Hospital, Boston, MA USA; 5 Department of Anesthesiology, Boston Medical Center, Boston, MA USA

**Keywords:** COVID-19, Anesthesia machine, ARDS, Mechanical ventilation, Intensive care unit

## Abstract

**Background:**

The surge of critically ill patients due to the coronavirus disease-2019 (COVID-19) overwhelmed critical care capacity in areas of northern Italy. Anesthesia machines have been used as alternatives to traditional ICU mechanical ventilators. However, the outcomes for patients with COVID-19 respiratory failure cared for with Anesthesia Machines is currently unknow. We hypothesized that COVID-19 patients receiving care with Anesthesia Machines would have worse outcomes compared to standard practice.

**Methods:**

We designed a retrospective study of patients admitted with a confirmed COVID-19 diagnosis at a large tertiary urban hospital in northern Italy. Two care units were included: a 27-bed standard ICU and a 15-bed temporary unit emergently opened in an operating room setting. Intubated patients assigned to Anesthesia Machines (AM group) were compared to a control cohort treated with standard mechanical ventilators (ICU-VENT group). Outcomes were assessed at 60-day follow-up. A multivariable Cox regression analysis of risk factors between survivors and non-survivors was conducted to determine the adjusted risk of death for patients assigned to AM group.

**Results:**

Complete daily data from 89 mechanically ventilated patients consecutively admitted to the two units were analyzed. Seventeen patients were included in the AM group, whereas 72 were in the ICU-VENT group. Disease severity and intensity of treatment were comparable between the two groups. The 60-day mortality was significantly higher in the AM group compared to the ICU-vent group (12/17 vs. 27/72, 70.6% vs. 37.5%, respectively, *p* = 0.016). Allocation to AM group was associated with a significantly increased risk of death after adjusting for covariates (HR 4.05, 95% CI: 1.75–9.33, *p* = 0.001). Several incidents and complications were reported with Anesthesia Machine care, raising safety concerns.

**Conclusions:**

Our results support the hypothesis that care associated with the use of Anesthesia Machines is inadequate to provide long-term critical care to patients with COVID-19. Added safety risks must be considered if no other option is available to treat severely ill patients during the ongoing pandemic.

**Clinical trial number:**

Not applicable.

**Supplementary Information:**

The online version contains supplementary material available at 10.1186/s12871-021-01376-9.

## Background

A major priority amid the emergency response to surges of coronavirus disease-2019 (COVID-19) cases has been to increase hospital capacity, particularly in terms of ICU bed availability [1]. In many hospitals, this undertaking has included converting operating rooms (OR) and post-anesthesia care units (PACU) into temporary ICUs [1, 2].

Using anesthesia machines in addition to standard ICU ventilators significantly increases a hospital’s capacity to provide critical ventilatory support. Several authors and societies have advocated using Anesthesia Machines in COVID-19 patients at institutions faced with resource limitations [3–5].

Critical care ventilators are designed to function as mostly unattended devices. Alarms are usually integrated with an overhead monitoring system and trigger personnel from a distance. Through sophisticated tools and software, different ventilatory modes can be applied in a wide array of critical respiratory conditions. Inspired gases are usually actively humidified, and exhaled breath is dispersed in the room air after filtration with a bacterial filter [6].

The ventilator apparatus attached to an anesthesia machine is designed to be closely attended by trained professionals in the OR. Anesthesia Machines usually provide ventilation only while the patient is unconscious and paralyzed for surgery, with a limited range of available ventilatory settings and monitoring features. Anesthesia Machine workstations can be used to deliver inhaled anesthetics through dedicated vaporizers. Dry compressed gases are passively humidified through a heat and moisture exchanger, usually with filtration properties. A unique feature of Anesthesia Machines is the ability to regulate an inlet of fresh gas flow, altering the amount of rebreathed exhaled gas via a scavenger system. While the use of filters and a closed system might be attractive options during the pandemic to limit viral contamination of the room and to spare medical gases, long-term use of Anesthesia Machines could also pose several complications [3, 7].

Overall, Anesthesia Machines can provide life-sustaining mechanical ventilation, but they were not originally designed to support critically ill patients for prolonged times [8]. At the start of the 2020 pandemic, a registry was formed to understand patterns and trends in the critical care being delivered to patients with COVID-19 requiring mechanical ventilation. In this retrospective observational study, we investigated how the care of patients who received Anesthesia Machines versus the care of patients who received ICU ventilators impacted mortality. We hypothesized that 60-day survival would be reduced in patients cared for with Anesthesia Machines compared to care that involved standard ICU ventilators.

## Methods

### Data source

The COVID-ICU multicenter registry is an international data repository that started on March 1st, 2020 and is currently ongoing. It includes de-identified daily data relative to critically ill patients with confirmed COVID-19 admitted to the ICU. The anonymized data collection strategy utilizes a secure cloud-based online platform (Studytrax, Macon, GA) [9]. The present study received approval by the Institutional Review Board of the coordinating institution (Massachusetts General Hospital, Boston, MA, USA, Protocol #2020P000760) with a waiver of informed consent. Patients included in this study were all admitted to Niguarda Hospital, Milan, Italy (Approval No.183–15,042,020).

### Identification of the study cohort

In the first week of March, due to an overwhelming need for ICU beds, Niguarda Hospital (Milan, Italy) converted a large postoperative 27-bed ICU and an OR in two COVID-19 specific ICUs. The former unit was a 27-bed standard ICU, fully equipped with state-of-the-art ICU ventilators and was used entirely for COVID-19 from March 12th to April 15th, 2020. The second unit was emergently opened (from March 5th to May 15th, 2020) in an adjacent OR area. This OR ICU consisted of a 15-bed temporary ICU, including five beds from a PACU space and 10 beds placed in five separate ORs (two beds per single OR). This provisional unit was equipped with seven Anesthesia Machines, alongside eight standard ICU ventilators. These two units were equipped with the same medical, nursing, and support staff who usually worked in the standard postoperative ICU. Physicians and nurses within both the standard ICU and the OR ICU were highly trained individuals in the field of anesthesia and critical care. Particularly, the OR ICU was equipped with nurses skilled in the use of standard ICU ventilators and Anesthesia Machines. To further improve the Anesthesia Machines management in the OR-ICU, we planned weekly training sessions on their use by an expert anesthesiologist and two expert nurses of anesthesia. Moreover, every morning an expert nurse of anesthesia and an Anesthesiologist revised the Anesthesia Machines in function (filters, circuits and ventilator) in order to improve patient’s safety.

During the study period, patients were admitted to either unit and assigned to an ICU ventilator, or an Anesthesia Machine, based on bed availability. Patients assigned to Anesthesia Machines were never switched from ICU ventilators and likewise switching did not occur for patients assigned to ICU ventilators.

### Study design

We designed a retrospective study comparing intubated COVID-19 patients whose care involved the use of Anesthesia Machine (AM group) to a cohort receiving care involving the use of standard ICU ventilators (ICU-VENT group) admitted during the same period. The “Strengthening the Reporting of Observational studies in Epidemiology” (STROBE) guidelines were followed [10].

Patients 18 years-or-older admitted to either the standard ICU or the OR ICU were included. A confirmed diagnosis of SARS-CoV-2 infection was required. Patients receiving less than 48-h of mechanical ventilation were excluded. The primary endpoint was to assess a difference in 60-day survival between the two groups. Secondary endpoints included evaluation of differences between AM and ICU-VENT groups in terms of ICU or hospital length of stay, ventilator-free days, ICU and hospital free days, need for ECMO, need for tracheostomy, incidence of barotrauma (defined as spontaneous pneumothorax or pneumomediastinum) and need for emergency endotracheal tube exchange secondary to airway occlusion. We defined ventilator-free days as the total number of calendar days or part calendar days of unassisted breathing during the first 60 days after ICU admission; all patients who died by day 60 were assigned zero ventilator-free days. ICU and Hospital free days were defined respectively as the total number of calendar days the patient spent out of the ICU or Hospital during the first 60 days after ICU admission. All patients who died by day 60 were assigned zero ICU or Hospital-free days. All outcomes and variables included in this analysis were abstracted from the medical record using clinically documented values.

### ICU care

Patients were treated according to internationally recognized standards of care [11]. Details regarding mechanical ventilation settings, the use of respiratory failure rescue strategies, and COVID-19 specific therapies are reported in the online supplement (See “ICU care and COVID-19 specific therapies” in Supplemental Digital Content 1).

### Anesthesia machine setup

The Primus™ workstation (Dräger, Lubeck, Germany) was the only Anesthesia Machine used. A heat and moisture exchanger with a filter (HMEF) and filter exclusive to the airway were used for every patient. The HMEF was placed at the endotracheal tube mouthpiece (DAR™ Adult-Pediatric Electrostatic Filter HME, Small, Medtronic, Minneapolis). The airway filter without HME (DAR™ Electrostatic Filter, Large, Medtronic, Minneapolis) was placed at the end of the expiratory limb of the circuit [8]. Both devices were routinely changed every 24 h or if there were signs of obstruction. For every Anesthesia Machine, a successful startup test was performed at baseline and at least every 72-h. A rebreathing circuit was in place, with a soda-lime scavenger to adsorb carbon dioxide. During approximately the first month of the study, the total fresh gas flow rate was maintained at 50–60% of the patient’s minute ventilation, with the intent to spare sevoflurane and oxygen. Following the publication of consensus recommendations, the fresh gas flow was increased to around 80% of minute ventilation in patients receiving halogenates, and to over 100% in patients without inhaled anesthetic [8, 12].

### ICU ventilators

The SERVO-i Mechanical Ventilator (Getinge, Gothenburg, Sweden) is the primary ICU ventilator in use at the study institution. A similar filtering strategy to that used in the AM group was initially implemented, with an HMEF and an exclusive airway filter placed at the endotracheal tube and expiratory inlet on the ventilator, respectively. However, as soon as adequate supplies of personal protective equipment and proper isolation logistics could be guaranteed, active humidification was preferred to HMEF for most patients in the ICU-VENT group.

### Statistical analysis

The statistical plan was written after the data were accessed and no statistical power calculation was performed prior to the start of the study. Instead, the sample size was based on all available data from the time period in which both units were functioning as COVID ICUs.

Baseline characteristics are presented as median and interquartile range for continuous covariates, and proportions for categorical variables. The Mann-Whitney U test or Fisher’s exact test were used for differences between the two groups (AM group vs. ICU-VENT group), and survivors vs. non-survivors, as appropriate. Kaplan-Meier survival analysis was used to compare 60-day survival between the two cohorts. Significance was assessed using a Log-Rank test. There were no censored survival data in this study.

In order to evaluate the impact of receiving care with an Anesthesia Machine on patient survival, we performed an adjusted and multivariable analysis. After confirming the proportional hazards assumption was met (*p* = 0.86), Cox Regression models were performed, in which we assessed the relationship with 60-day mortality. In the adjusted model only variables with *p* < 0.10 on the univariate screen were considered candidate variables for inclusion. Using this list of variables, backwards selection was then performed (considering *p* < 0.1 for exclusion at each step) in order to elucidate a final model. Hazards ratios (HR) and their associated 95% confidence intervals (CI) from the final model are presented. SPSS software v26 (Microsoft Corporation – Redmond, USA) and SAS 9.4 (SAS Institute Inc., Cary, NC) was used for data analysis. Two-sided *p*-values < 0.05 were considered statistically significant.

A sensitivity analysis was performed in order to address the possibility of biased estimates of the predictors by using a data-driven variable selection process. In this analysis 100 bootstrapped samples were obtained from the original dataset with replacement, and backward selection was similarly performed as above for each of the bootstrapped samples. Variables in the final sensitivity model were based on frequency and included the four most common variables in order to maintain model parsimony. Full results of the model selection process are detailed in Supplementary Digital Content 2 Table 4.

Rates of missing data are reported in Supplementary Digital Content 2 Table 3. No imputation was performed for missing data.

## Results

From March 5 to May 15, 2020, a total of 156 critically ill COVID-19 patients have been treated at Niguarda Hospital. Among these, 93 patients were admitted to the two study units: 52 to the standard ICU and 41 to the OR ICU. Four patients were excluded from the analysis (three patients did not need invasive mechanical ventilation; one patient was transferred within 24 h). Eighty-nine patients were included in the study. Most of the patients in both groups (AM group and ICU-VENT group) were admitted to the ICUs during the month of March: 16 (94%) for the AM group and 58 (81%) for the ICU-VENT group (*p* = 0.28).

For 17 patients (19%), an Anesthesia Machine was used (AM group), while for 72 (81%), an ICU ventilator was available (ICU-VENT group). Baseline characteristics are reported in Table 1. The only difference of note between groups with respect to staffing was that patients in the OR ICU had a slightly higher nurse-to-patient ratio. (See “ICU Staffing” in Supplemental Digital Content 1).

**Table 1:** Baseline characteristics of the study population

	Overall (*n* = 89)	Anesthesia Machine (*n* = 17)	ICU ventilator (*n* = 72)	*P* value
Age, yr	59 [51–67]	62 [53–70]	59 [51–66]	0.333
Male sex, no. (%)	70 (78.7)	12 (70.6)	58 (80.6)	0.510
BMI, kg/m^2^	28 [26–31]	27 [25–30]	28 [26–32]	0.262
*Race, no. (%)*				0.657
White	73 (82.0)	13 (76.4)	60 (83.3)	
Black	2 (2.2)	1 (5.8)	1 (1.3)	
Asian	2 (2.2)	0 (0)	2 (2.7)	
Other	12 (13.4)	3 (17.6)	9 (12.5)	
*Severity at admission*				
APACHE II score	11 [8–14]	14 [8–16]	11 [8–14]	0.133
SOFA score	5 [3–7]	6 [4–8]	4 [3–7]	0.188
*Comorbidities*, no. (%)				
Hypertension	46 (51.7)	7 (41.2)	39 (54.2)	0.422
Diabetes mellitus	16 (18.2)	3 (17.7)	13 (18.3)	1.000
Obesity	25 (28.4)	3 (17.7)	22 (31.0)	0.375
COPD	5 (5.6)	1 (5.9)	4 (5.6)	1.000
Hypercholesterolemia	15 (16.9)	3 (17.7)	12 (16.7)	1.000

Data reported as Number (Percentage) or Median [Interquartile Range]; Differences between groups were assessed with a Mann-Whitney U test or Fisher’s Exact test depending on variable type. BMI: Body Mass Index; APACHE: Acute Physiology and Chronic Health Evaluation; SOFA: Sequential Organ Failure Assessment; COPD: Chronic Obstructive Pulmonary Disease

### Patients in the AM group showed similar COVID-19 severity at admission compared to the ICU-VENT group (Table 2)

The degree of respiratory impairment and mechanical ventilation requirement were similar between the two cohorts. Although not statistically significant, the AM group showed slight lower levels of Mean Arterial Pressure at ICU admission (71 [67–76] mmHg vs. 78 [70–86] mmHg; AM vs. ICU-VENT group, respectively; *p* = 0.050). No significant differences in mean arterial pressure were observed after day one between the ICU-VENT and AM groups (See Figure 1 in Supplemental Digital Content 2).

**Table 2:** Severity of disease at ICU admission and treatment received in the ICU

	Overall (*n* = 89)	Anesthesia Machine (*n* = 17)	ICU Ventilator (*n* = 72)	*P* value
**Clinical variables**				
PaO_2_/FiO_2_, mmHg	172 [126–219]	197 [136–221]	170 [123–208]	0.204
PEEP, cmH_2_O	14 [12–14]	12 [12–14]	14 [12–14]	0.181
P_plat_, cmH_2_O	24 [22–26]	24 [21–26]	24 [22–26]	0.838
Vt/PBW (ml/kg)	6 [6–7]	6.1 [5.7–6.5]	6.4 [6.0–7.2]	0.042
RR, breaths/min	20 [18–22]	20 [18–22]	20 [18–22]	0.795
C_rs_, ml/cmH_2_O	44 [36–53]	38 [32–44]	45 [37–53]	0.118
dP, cmH_2_O	10 [8–12]	11 [8–12]	10 [8–12]	0.854
PaO_2_, mmHg	92 [82–115]	94 [84–110]	92 [82–117]	0.951
PaCO_2_, mmHg	47 [41–56]	47 [43–57]	46 [41–54]	0.382
pH	7.37 [7.30–7.40]	7.36 [7.30–7.41]	7.37 [7.30–7.39]	0.854
HCO_3_^−^, mmol/l	26 [24–28]	25 [25–27]	26 [24–28]	0.897
Base excess	0 [−2, 2]	1 [−2, 3]	0 [−2, 2]	0.465
Lactate, mmol/l	1.2 [1.0–1.6]	1.5 [1.2–1.8]	1.2 [1.0–1.5]	0.017
Heart rate, beats/min	80 [66–92]	68 [62–85]	80 [68–92]	0.132
MAP, mmHg	77 [70–85]	71 [67–76]	78 [70–86]	0.050
**Laboratory findings**				
CRP, mg/dl	12.4 [7.2–17.8]	8.8 [6.8–15.3]	12.5 [7.8–21.1]	0.241
Procalcitonin, ng/ml	0.4 [0.2–0.9]	0.5 [0.2–1.0]	0.4 [0.2–0.8]	0.753
WBC, 10^9^/L	8.9 [6.7–12.2]	9.1 [7.2–12.5]	8.9 [6.4–12.1]	0.689
Tot. lymphocytes, %	8.1 [4.9–12.2]	8.8 [4.9–11.2]	8.0 [4.9–12.5]	0.955
Hematocrit, %	38 [34–41]	38 [35–41]	38 [34–41]	0.946
Hemoglobin, mg/dl	13 [11–13]	13 [12–13]	13 [11–13]	0.837
Platelets, 10^12^/L	230 [180–289]	214 [172–290]	232 [185–288]	0.807
ALT, IU/L	39 [26–53]	41 [35–51]	35 [25–58]	0.309
AST, IU/L	41 [29–61]	45 [35–73]	40 [29–61]	0.344
LDH, IU/L	425 [356–544]	425 [356–559]	428 [356–535]	0.827
Bilirubin, mg/dl	0.7 [0.5–1.1]	0.9 [0.5–1.9]	0.7 [0.5–1.1]	0.241
Creatinine, mg/dl	0.9 [0.7–1.0]	1.0 [0.8–1.1]	0.8 [0.7–1.0]	0.171
Glucose, mg/dl	131 [112–167]	143 [123–161]	130 [110–171]	0.446
Sodium, mEq/l	137 [134–140]	139 [136–141]	137 [133–139]	0.080
Potassium, mEq/l	4.1 [3.7–4.4]	4.1 [3.7–4.5]	4.1 [3.7–4.4]	0.972
Creatine kinase, IU/l	117 [67–180]	119 [38–136]	117 [67–200]	0.407
CK-MB, ng/ml	1.3 [0.9–2.9]	1.1 [0.9–1.7]	1.5 [1.0–3.0]	0.184
Troponin-T, ng/l	14 [8–29]	9 [6–22]	15 [8–39]	0.313
NT-proBNP, ng/l	335 [125–746]	421 [230–818]	289 [125–746]	0.682
PT, s	14 [14–15]	15 [14–17]	14 [14–15]	0.059
aPTT, s	36 [32–42]	38 [33–44]	36 [32–42]	0.501
D-dimer, mcg/ml	3.4 [0.9–10.3]	8.7 [2.2–35]	2.1 [0.8–7.3]	0.073
**Treatments received, no. (%)**				
Lopinavir/ritonavir	58 (64.4)	14 (82.3)	44 (61.1)	0.156
Hydroxychloroquine	75 (83.3)	16 (94.1)	59 (81,9)	0.290
Antibiotic prophylaxis	24 (26.7)	8 (47.0)	16 (22.2)	0.065
Corticosteroids	32 (36.4)	6 (35.3)	26 (36.6)	1.000
Tocilizumab	30 (34.1)	5 (29.4)	25 (35.2)	0.780
Remdesivir	10 (11.4)	1 (5.9)	9 (12.7)	0.679
Continuous IV sedation	64 (71.9)	3 (17.7)	61 (84.7)	< 0.001
Continuous IV opioids	61 (67.8)	12 (70.5)	49 (68.0)	0.544
Inhaled sedation	14 (15.7)	14 (82.3)	0 (0)	< 0.001
Paralysis	74 (82.2)	16 (94.2)	58 (80.5)	0.285
Prone positioning	44 (50.0)	11 (64.7)	33 (46.5)	0.280
Inhaled Nitric Oxide	10 (11.4)	3 (17.7)	7 (9.9)	0.399
ECMO	6 (6.7%)	0 (0)	6 (8.3)	0.591

Data reported as Number (Percentage) or Median [Interquartile Range]; Differences between groups were assessed with a Mann-Whitney U test or Fisher’s Exact test depending on variable type. Treatments received: Therapy administered for one or more ICU day(s); PEEP: Positive End-Expiratory Pressure; P_plat_: Plateau Pressure; Vt: Tidal Volume; PBW: Predicted Body Weight; RR: Respiratory Rate; C_rs_: Respiratory System Compliance; dP: Driving Pressure (P_plat_ – PEEP); MAP: Mean Arterial Pressure; CRP: C-Reactive Protein; WBC: White Blood Cells; ALT: Alanine Aminotransferase; AST: Aspartate Aminotransferase; LDH: Lactate Dehydrogenase; NT-proBNP: N-Terminal pro B-type Natriuretic Peptide; PT: Prothrombin time; aPTT (activated partial thromboplastin time); ECMO: extra corporeal membrane oxygenator

### The intensity of treatment and the use of rescue therapies were comparable between the two groups (Tables 2 and 3)

Mechanical ventilation settings were similar in the AM and ICU-VENT group. All patients received prolonged mechanical ventilation, with no difference between the AM and ICU-VENT groups (12 [4–28] days vs. 14 [10–27] days, AM vs. ICU-VENT group, respectively; *p* = 0.364). Forty-four patients received at least one cycle of prone positioning during their ICU stay, with pronation rates similar between groups (64.7% vs. 46.5%, AM vs. ICU-VENT group, respectively; *p* = 0.280). Inhaled nitric oxide was administered once or more during ICU stay to a total of 10 patients at an average concentration of 40 ppm for 48 h (17.7% vs. 9.9%, AM vs. ICU-VENT group, respectively; *p* = 0.399). Six patients, all in the ICU-VENT group (8.3%), underwent VV-ECMO support (*p* = 0.591).

**Table 3:** 60-Day Crude Outcome Differences

	Overall (*n* = 89)	Anesthesia Machine (*n* = 17)	ICU Ventilator (*n* = 72)	*P* value
Mortality, no. (%)	39 (43.8)	12 (70.6)	27 (37.5)	0.016
ICU length of stay, days	*14 [9–33*]	12 [5–28]	16 [10–33]	0.280
ICU-free days	*0 [0–42]*	0 [0–6]	11 [0–45]	0.084
Hospital length of stay, days	26 [13–52]	19 [8–46]	26 [16–56]	0.122
Hospital-free days	0 [0–21]	0 [0–0]	0 [0–31]	0.104
Mechanical ventilation days	14 [9–279]	12 [4–28]	14 [10–27]	0.364
Ventilator-free days	11 [0–43]	0 [0–9]	21 [0–46]	0.033
ECMO initiation, no. (%)	6 (6.7)	*0 (0)*	6 (8.3)	0.591
Tracheostomy, no. (%)	42 (47.2)	9 (52.9)	33 (45.8)	0.788
Barotrauma ^a^, no. (%)	4 (4.5)	2 (11.8)	2 (2.8)	0.163
Emergency tube exchange ^b^, no (%)	4 (4.5)	3 (17.7)	1 (1.4)	0.021

Data reported as Number (Percentage) or Median [Interquartile Range]; Differences between groups were assessed with a Mann-Whitney U test or Fisher’s Exact test depending on variable type. ECMO: Extra-Corporeal Membrane Oxygenation; ICU: Intensive Care Unit; ^a^ Barotrauma defined as spontaneous pneumothorax and/or pneumomediastinum during mechanical ventilation; ^b^ Extubation and immediate re-intubation due to life-threatening airway occlusion

Patients in the AM group were more frequently treated, for 1 day or more, with inhalation anesthetics (0% vs. 82%, *p* < 0.001). In contrast, continuous intravenous (IV) sedation was used more often in the ICU-VENT group (84.7% vs. 17.7%, *p* < 0.001).

### Critical COVID-19 patients in the AM group died more frequently compared to those in the ICU-VENT group (Table 3 and Fig. 1)

**Fig. 1:**
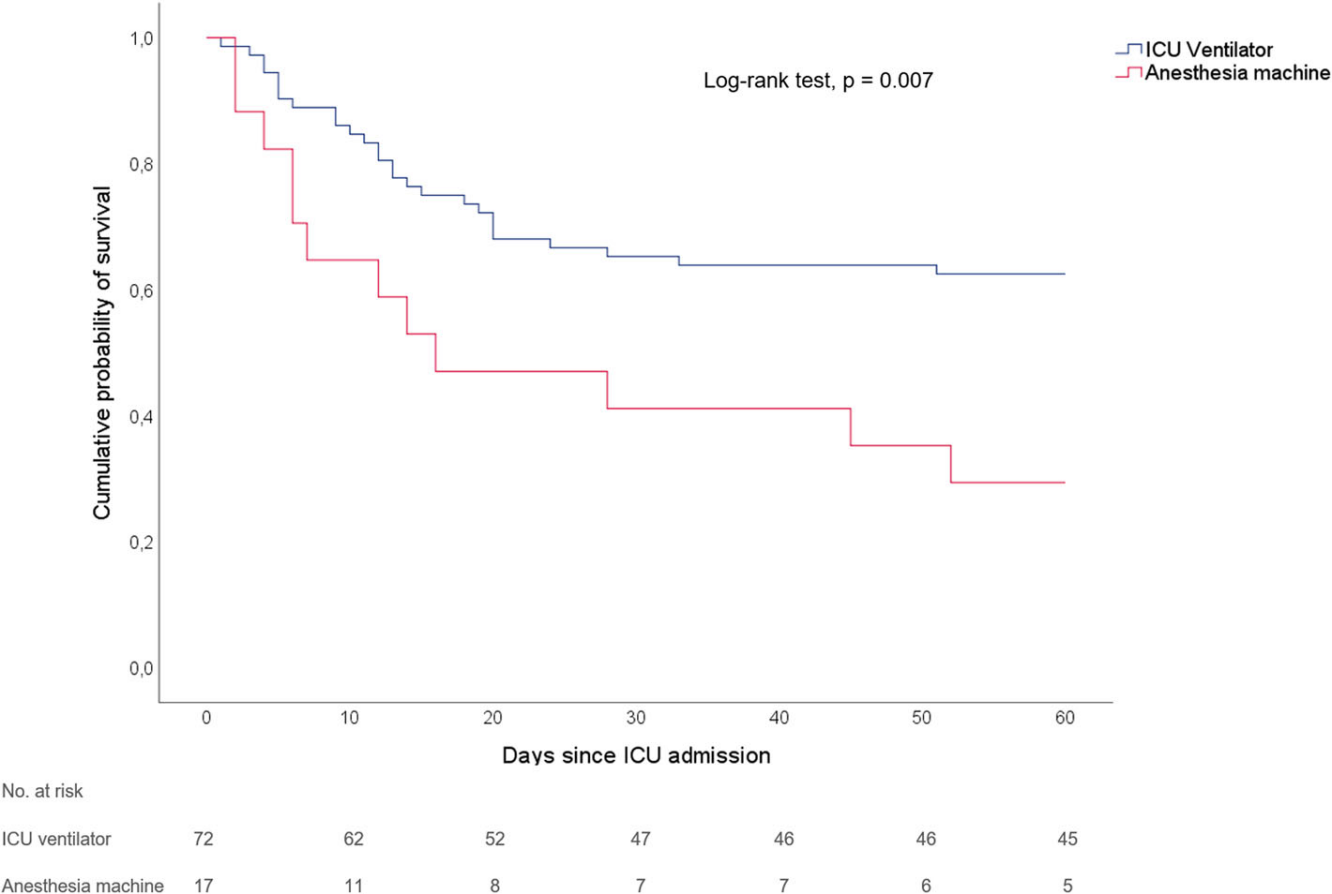
Kaplan-Meier curve of 60-days survival for patients allocated to ICU ventilators and Anesthesia Machines

The overall 60-day mortality was 43.8% (39/89 patients). Comparing study groups, patients in the AM group experienced a remarkably reduced 60-day survival (deaths: 12/17 vs. 27/72; mortality rate: 70.6% vs. 37.5%, AM group vs. ICU-VENT group, respectively; *p* = 0.016). Figure 1 reports the Kaplan-Meier Survival curve at 60-days (log-rank *p* = 0.007). Both the ICU and hospital length-of-stay did not differ between the two groups.

Comparing the two ICUs we report a 60-day mortality of 51.2% in the OR ICU and of 37.5% in the conventional-ICU (p = 0,207). Moreover, the 60-day mortality among patients receiving ICU ventilator care (regardless of ICU setting) was identical (37,5%).

Table 1 of Supplemental Digital Content 2 details the causes of death among the two groups.

### Care of patients that involved the use of anesthesia machines was independently associated with an increased risk of death, adjusting for potential confounding factors in a multivariable regression model (Table 4)

In a univariate analysis (Table 2 in Supplemental Digital Content 2), 60-day mortality was significantly higher in patients who at baseline had the following characteristics: were cared for with Anesthesia Machines, were older, had a higher body mass index, showed higher lactate, lower driving pressure, lower pH, higher hemoglobin, higher bilirubin, higher creatinine level, or those with a history of hypertension, diabetes, COPD or hypercholesterolemia. A higher mean arterial pressure was associated with a limited, although significant, protective effect (HR 0.96 per mmHg, 95% CI 0.93–0.99, *p* = 0.008). Of note, we performed an analysis of blood pressure trends over the course of several ICU days (days 1, 2, and 7) among patients receiving volatile anesthetic and found no evidence suggesting lower blood pressures in this subgroup (see Figure 1 in Supplemental Digital Content 2).

**Table 4:** Multivariable Cox Regression Analysis of Hazard Ratios for 60-Day Mortality

	Hazard Ratio	95% CI	*P* value
Allocation to anesthesia machine	4.05	1.75–9.33	0.001
Age at admission, per year	1.08	1.02–1.13	0.004
Bilirubin, per mg/dl	1.45	1.11–1.90	0.007
Creatinine, per mg/dl	7.20	2.57–20.21	< 0.001
Diabetes Mellitus	4.02	1.63–9.91	0.003

*95% CI* 95% Confidence Interval

After adjustment for confounders, care that involved the use of an AM was associated with a significantly increased risk of death at 60 days (HR 4.05, 95% CI 1.75–9.33, *p* = 0.001). Other variables associated with increased risk of death at 60 days after adjustment for confounders included: older age (HR 1.08 per year, 95% CI 1.02–1.13, *p* = 0.004), creatinine (HR 7.20 per mg/dl, 95% CI 2.57–20.21, *p* < 0.001), bilirubin levels (HR 1.45 per mg/dl, 95% CI 1.11–1.90, *p* = 0.007) at ICU admission, and a history of diabetes (HR 4.02, 95% CI 1.63–9.91, *p* = 0.003; Table 4).

In a sensitivity model in which model covariates were based on inclusion frequency in a bootstrapped sample, the final model included the covariates for creatinine, hypertension and bilirubin in addition to the use of anesthesia machines. In this adjusted model the association between the use of anesthesia machines and 60-day mortality remained robust (HR 3.46, 95% CI 1.57–7.63, *p* = 0.002; Supplementary Digital Content 2 Tables 4 and 5).

### The use of anesthesia machines for prolonged periods might be associated with the risk of technical failure or airway occlusion (Table 5)

During the study period, two cases of sudden Anesthesia Machine failure were observed that required emergent replacement of the workstation. No technical issues were experienced in the ICU-VENT group. Several episodes of mucus plugging of the endotracheal tube occurred in the AM group. In most instances, the obstruction resolved with vigorous suction, or fiberoptic bronchoscopy. Emergency tube exchange was needed in 3/17 cases (18%), compared to 1/72 (1%) in the ICU-VENT group (*p* = 0.021). One patient in the AM group died due to sudden complete airway obstruction following the accumulation of secretions at the level of the carina, which could not be effectively and timely relieved.

**Table 5:** Issues encountered with the use of anesthesia machines in COVID-19 critically patients and relative proposed solutions

Problem	Proposed solution
Audibility and correct perception of alarms potentially associated with life-threatening AM failures	• Constant presence of anesthesia providers in the clinical team; • Maximize staff proximity to the workstation.
Condensed water accumulation in the circuit causing obstruction of HMEF or filters Reduced reliability of flow sensors	• Use of high fresh gas flow (dryer gas mixture); • HME perpendicularly positioned above the endotracheal tube to reduce the backflow of excess moisture into the circuit; • Use of heated breathing circuits, condensers and water traps to limit water accumulation.
Endotracheal tube obstruction	• Dedicated endotracheal tube cleaning devices.
Frequent disconnection due to filter change and machine self-tests	• Temporary use of a portable ventilator during disconnection to maintain protective ventilation and PEEP settings.
Limited functionality for the assessment of respiratory mechanics	• Prioritize the use of newer AMs in more complicated patients considering the possibility to perform measurements of respiratory mechanics (e.g. end-inspiratory and end-expiratory pauses).

*HMEF* Heat and Moisture Exchanger with Filter, *PEEP* Positive End-Expiratory Pressure, *AM* Anesthesia Machine

## Discussion

This is the first analysis of a relatively large registry investigating mortality in patients who received care with Anesthesia Machines versus standard ICU ventilators for COVID-19 respiratory failure during a peak surge. Our results indicate that during the emergency response to the initial peak of COVID-19, the care of critically ill patients with repurposed Anesthesia Machines was associated with an increased rate of complications and mortality.

Italy was the first country outside of China to suffer a major outbreak of SARS-CoV-2 infection and the registry population provided unique advantages for studying the impact of Anesthesia Machines use in the care of COVID-19 patients compared to standard ICU-VENT use. The population we described in this study had similar characteristics to that of other published case series [13–17]. The 70% mortality for patients in the AM group however is remarkably high. The overall 60-day mortality though of the analyzed cohort (43.8%) is similar to what has recently been reported by the COVID-19 Lombardy ICU Network in the largest currently available outcome study of Italian cases (48.7%) [14]. While greatly varying across the multiple available reports, ICU mortality from COVID-19 is primarily driven by the development of ARDS, with 50% mortality among patients with COVID-19 ARDS generally considered an accepted estimate [18]. Our overall mortality findings are, therefore, in agreement with the currently available literature, supporting the quality and external validity of our data.

Given the dramatic increase in mortality that we observed in patients whose care involved the use of Anesthesia Machine, we aimed to quantify factors associated with their care contributing to lethality. We wish to be explicit in stating that our registry analysis does not allow us to conclude that the Anesthesia Machine ventilator itself is the exclusive culprit. Instead, we believe that our study demonstrates that the clinical care scenarios associated with using Anesthesia Machines are linked to increased mortality. There are several considerations regarding possible changes in clinical care and unique Anesthesia Machine-related challenges that should be discussed.

First, the correct setup of audible alarms on Anesthesia Machines and the ability to respond with a prompt corrective action might prove challenging for any operator, particularly when clinicians are trying to limit proximity to patients and must don and doff personal protective equipment [5, 8]. We believe that several non-quantifiable factors related to Anesthesia Machines not being a normal standard of *ICU* care – even for clinicians comfortable with their operation in the OR setting – could have led to a higher degree of mortality.

Second, clogging of HMEF and filters due to excess moisture or secretion burden is a major problem in patients receiving prolonged ventilation on an AM without a heat source and active humidification, particularly when low fresh gas flow is used [19, 20]. HMEs are a passive form of humidification. The device stores heat and moisture from the patient’s own exhaled gas which is released during inhalation of fresh gas, which would otherwise be dry and at ambient temperature [21, 22]. In our study, frequent HME replacements were required in the AM group to prevent occlusion. The use of a higher fresh gas flow rates reduced this complication and is currently recommended by the APSF/ASA guidelines [8].

Third, COVID-19 patients often show tenacious and abundant tracheal secretions, whose inspissation might lead to an even higher risk of ETT occlusion. Given the lower temperature in the Anesthesia Machine circuit (without a dedicated heating system or active humidification), we believe Anesthesia Machines could make this risk even higher. In COVID-19 patients ventilated with repurposed Anesthesia Machines, Panchami, KR, et al. reported a 29% incidence of critical airway obstruction requiring emergency Fiber Optic Bronchoscopy or tube exchange [23]. In our study, subtotal tube obstruction due to mucus accumulation was also a common occurrence with Anesthesia Machines. In one case, an exceptionally large mucus plug caused a sudden complete airway obstruction at the level of the carina leading to hypoxia and cardiac arrest. It reasonable to presume that inadequate heating and humidification could also have led to increased secretion burden and decreased secretion clearance in more distal airways.

Four, we estimate that each patient on an Anesthesia Machine had to be disconnected roughly twice per day on average, to either change filters, or perform startup self-tests. On these occasions, we had no other choice than to ventilate the patient with a manual resuscitator. Disconnections from the mechanical ventilator in ARDS might result in loss of PEEP and lung collapse and should be avoided at all costs [24, 25]. The use of manual bag ventilation might lead to hyperventilation with excessive rate, pressure, and tidal volume, all critical determinants of VILI [26, 27].

Finally, one must theoretically consider that ventilator-induced lung injury of increased severity is also a potential explanation for the decreased survival in patients in the AM group. The accumulation of excess condensation in the circuit of AMs often hindered the accuracy of flow sensors and increased resistance, leading to inconsistently delivered tidal volumes. The importance of an accurately set tidal volume within a lung-protective ventilatory strategy is a mainstay of ARDS treatment [28, 29].

All the points discussed above might also explain themselves a lower amount of Ventilator free-days observed in the AM-group, however we believe that it mainly reflect a higher mortality rate in this group.

Our registry demonstrated a significantly increased use of volatile anesthetic in patients receiving Anesthesia Machines. We believe the OR ICU in this study represented the safest possible setting for volatile anesthetic use given the presence of experienced personnel, proper monitoring systems and adequate machines. For these reasons we largely utilized Sevoflurane as the main sedative drug the critically ill phase of our patients undergoing mechanical ventilation with the AMs. Therefore, the AM group cohort had significantly more volatile anesthetic use compared to the ICU-VENT group. We do not have a high suspicion that the use of halogenates could explain the difference in outcome between the two groups for the following reasons. The use of sevoflurane as a first-line sedative drug in ARDS patients has shown shorter awakening and extubation times compared to IV sedatives [30]. Additionally, Jabaudon et al. observed that when Sevoflurane was compared with midazolam in ARDS patients, oxygenation improved, and alveolar/systemic inflammation and lung epithelial injury were reduced [31]. For these reasons some authors have even suggested that inhaled volatile agents may provide important pulmonary benefits for COVID-19 patients with ARDS [32, 33]. We were more concerned that there could be a potential connection between lower blood pressures and inhaled anesthetic use. However, an analysis of blood pressures amongst groups during the first week of ventilation did not reveal concerning trends (Figure 1 in Supplemental Digital Content 2).

The allocation to repurposed AM was not the only factor associate with higher risk of 60-day mortality. Higher age, history of diabetes mellitus and a severer organ damage (measured by creatinine and bilirubin) at ICU admission are also predictors for worst outcome. These findings are in agreement with currently available literature on critical COVID-19 [13, 14, 17].

Although our initial experience using Anesthesia Machines in the COVID-19 pandemic saw an increased incidence of mortality, we do hope that there were learned lessons that we can share with other clinicians currently experiencing COVID-19 surges. In the event that Anesthesia Machines are required to keep hospital capacity afloat, a summary of the issues we encountered using Anesthesia Machines and related proposed solutions is provided in Table 5.

### Limitations

Our study presents several limitations. Our analysis sought to limit confounding factors and to study the use of Anesthesia Machines as the only difference between groups. However, our sample size is relatively small. It is possible because of the small group size there are differences that persist between groups that were not identified as statistically significant in our analysis, but may be clinically meaningful. As in any retrospective study there is the possibility of residual confounding or bias in our interpretation. It should be noted however that patients who received AMs did so because of bed availability, not patient acuity or other factors, thus these are not likely biasing our results. Detailed data on bed assignments are not available. Our study took place during the very early stages of the pandemic. At the time, we witnessed a shifting emphasis on using certain drugs (e.g., antivirals, hydroxychloroquine, or immunomodulators). Their use in our cohort has been fragmented and based on institutional indications and local availability, rather than supporting evidence. Globally, a better understanding of the disease became available in the summer months of 2020. Our group gained growing experience in the management of COVID-19 patients as time went by, raising the possibility that the results were influenced by secular trends. Finally given that the nature of the study is an analysis of a registry, we are not able to draw causal interpretations to our results. Rather, our results are suggestive of a pattern increased mortality associated with the clinical care of COVID-19 patients whose management involved Anesthesia Machines.

## Conclusion

Our analysis demonstrated a significantly increased risk of death in those patients whose care involved the use of Anesthesia Machines during a pandemic surge.

## Supplementary Information

**Additional file 1.**

**Additional file 2.**

## Data Availability

The dataset used during the current study is available from the corresponding author on reasonable request.
