# Supplementary material for: The repurposed use of anesthesia machines to ventilate critically ill patients with coronavirus disease 2019 (COVID-19)
Source: BMC Anesthesiol. 2021 May 20;21:155. doi: 10.1186/s12871-021-01376-9 (PMC8134805; doi:10.1186/s12871-021-01376-9)
Supplement: Supplementary file 2 — Additional file 2. [file 12871_2021_1376_MOESM2_ESM.docx]

**Supplemental Digital Content 2**

**Table 1: Cause of Death**

|  |  | | |  |  |  |  |  |  |  |  |
| --- | --- | --- | --- | --- | --- | --- | --- | --- | --- | --- | --- |
| **Patient** | | **AM** | **Age,** *years* | | **Sex** | **BMI** | **APACHE II** | **Barotrauma during mechanical ventilation^¶^** | **Acute Airway Obstruction during mechanical ventilation^§^** | **ICU Length of Stay,** *days* | **Main Cause of Death** |
| 1 | | Yes | 65 | | M | 32 | 14 | No | No | 6 | Refractory Septic Shock |
| 2 | | Yes | 71 | | M | 29 | 16 | No | No | 4 | Refractory Hypoxia |
| 3 | | Yes | 62 | | F | 28 | 9 | No | No | 45 | Refractory Hypoxia |
| 4 | | Yes | 65 | | F | 26 | 19 | No | No | 7 | Acute Pulmonary Thromboembolism |
| 5 | | Yes | 71 | | M | 28 | 14 | No | No | 3 | Refractory Hypoxia |
| 6 | | Yes | 50 | | M | 25 | 17 | No | Yes | 53 | Refractory Hypoxia |
| 7 | | Yes | 70 | | M | 28 | 17 | Yes | No | 17 | Refractory Hypoxia |
| 8 | | Yes | 64 | | M | 26 | 18 | No | No | 8 | ARDS / Bacterial Infection |
| 9 | | Yes | 40 | | M | 24 | 4 | No | Yes | 28 | ARDS / Bacterial Infection |
| 10 | | Yes | 73 | | F | 35 | 15 | No | No | 12 | Ischemic Bowel Disease |
| 11 | | Yes | 70 | | M | 24 | 14 | Yes | No | 6 | ARDS / Septic Shock |
| 12 | | Yes | 59 | | M | 26 | 12 | No | Yes | 14 | Acute Airway Obstruction |
| 13 | | No | 49 | | M | 26 | 8 | No | No | 9 | Refractory Septic Shock |
| 14 | | No | 68 | | M | 22 | 18 | No | No | 18 | Refractory Hypoxia |
| 15 | | No | 59 | | M | 25 | 7 | No | No | 18 | ARDS / Right Ventricular Failure |
| 16 | | No | 67 | | M | 27 | 14 | No | No | 12 | Refractory Hypoxia |
| 17 | | No | 68 | | M | 31 | 10 | No | No | 10 | ARDS / Bacterial Infection |
| 18 | | No | 62 | | F | 26 | 12 | No | No | 6 | Refractory Hypoxia |
| 19 | | No | 75 | | M | 44 | 26 | No | No | 7 | Refractory Hypoxia |
| 20 | | No | 59 | | M | 40 | 11 | No | No | 20 | Refractory Septic Shock* |
| 21 | | No | 64 | | M | 26 | 13 | No | No | 33 | Refractory Hypoxia |
| 22 | | No | 72 | | M | 31 | 15 | No | No | 3 | Refractory Hypoxia |
| 23 | | No | 63 | | M | 28 | 13 | No | No | 13 | Refractory Septic Shock |
| 24 | | No | 68 | | M | 31 | 17 | No | No | 13 | ARDS / Bacterial Infection |
| 25 | | No | 64 | | M | 26 | 13 | No | No | 20 | Refractory Septic Shock |
| 26 | | No | 54 | | M | 31 | 12 | No | No | 58 | Refractory Hypoxia |
| 27 | | No | 55 | | M | 25 | 28 | No | No | 12 | Refractory Septic Shock |
| 28 | | No | 61 | | M | 40 | 11 | No | No | 9 | Refractory Hypoxia |
| 29 | | No | 67 | | M | 35 | 11 | No | No | 6 | Refractory Septic Shock |
| 30 | | No | 27 | | M | 33 | 5 | No | No | 19 | Refractory Hypoxia |
| 31 | | No | 73 | | M | 28 | 11 | No | No | 20 | Refractory Hypoxia / Myocardial Infarction |
| 32 | | No | 64 | | F | 35 | 9 | Yes | No | 24 | Refractory Hypoxia |
| 33 | | No | 62 | | M | 28 | 20 | No | No | 5 | Multiple Organ Failure |
| 34 | | No | 70 | | M | 31 | 14 | No | No | 5 | Refractory Hypoxia |
| 35 | | No | 58 | | M | 32 | 5 | No | No | 4 | Refractory Hypoxia |
| 36 | | No | 70 | | M | 43 | 16 | No | No | 5 | Refractory Hypoxia |
| 37 | | No | 65 | | M | 24 | 13 | No | No | 11 | Refractory Hypoxia |
| 38 | | No | 53 | | M | 28 | 6 | No | No | 15 | Refractory Hypoxia |
| 39 | | No | 33 | | M | 36 | 8 | No | No | 14 | Refractory Septic Shock |

***Abbreviations****: AM = Allocation to Anesthesia Machine; BMI = Body Mass Index; APACHE II = Acute Physiology and Chronic Health Disease Classification System II; ICU = Intensive Care Unit; ARDS = acute respiratory distress syndrome.*

*¶ Barotrauma has been defined as* *spontaneous pneumothorax and/or pneumomediastinum during invasive mechanical ventilation.*

*§ Extubation and immediate re-intubation due to life-threatening airway occlusion*

**Patient Died After ICU discharge*

**Table 2: Univariate Cox Regression Analysis for 60-day Mortality**

| Variable | Hazard ratio | 95% Confidence Interval | P value |
| --- | --- | --- | --- |
| Female sex | 0.46 | 0.18-1.17 | 0.104 |
| Anesthesia machine^§^ | 2.46 | 1.25-4.87 | 0.010 |
| Age at admission, per year^§^ | 1.06 | 1.03-1.10 | 0.001 |
| BMI, per kg/m^2^ | 1.04 | 0.98-1.10 | 0.198 |
| Hypertension^§^ | 2.34 | 1.20-4.56 | 0.013 |
| Diabetes mellitus^§^ | 2.29 | 1.11-4.72 | 0.024 |
| Obesity | 1.59 | 0.83-3.06 | 0.165 |
| COPD^§^ | 5.85 | 2.23-15.30 | < 0.001 |
| Hypercholesterolemia^§^ | 2.08 | 0.98-4.38 | 0.055 |
| Bilirubin, per mg/dl^§^ | 1.48 | 1.18-1.86 | 0.001 |
| Creatinine, per mg/dl^§^ | 4.67 | 2.33-9.35 | < 0.001 |
| Lactate, per mmol/L^§^ | 1.96 | 1.35-2.84 | < 0.001 |
| D-dimer, per mg/dl | 1.02 | 0.99-1.05 | 0.115 |
| Mean arterial pressure, per mmHg^§^ | 0.96 | 0.93-0.99 | 0.008 |
| PEEP, per cmH_2_O | 1.12 | 0.97-1.31 | 0.133 |
| P_plat,_ per cmH_2_O | 0.94 | 0.85-1.05 | 0.272 |
| Driving Pressure, per cmH_2_O^§^ | 0.89 | 0.78-1.01 | 0.071 |
| Tidal Volume/PBW, per ml/kg | 0.81 | 0.53-1.22 | 0.308 |
| RR, per breath | 1.02 | 0.94-1.12 | 0.596 |
| PaO_2_, per mmHg | 1.00 | 0.99-1.01 | 0.729 |
| PaCO_2_, per mmHg | 1.02 | 0.99-1.04 | 0.165 |
| pH, per unit^§^ | 0.02 | 0.00-0.44 | 0.015 |
| Heart rate, per beat | 0.99 | 0.98-1.01 | 0.521 |
| WBC, per 10^9^/L | 0.99 | 0.93-1.05 | 0.674 |
| Hematocrit, per %^§^ | 1.11 | 1.04-1.19 | 0.003 |
| Hemoglobin, per mg/dl^§^ | 1.37 | 1.11-1.68 | 0.003 |
| Platelets, per 10^12^/L | 1.00 | 0.99-1.00 | 0.155 |
| APACHE II score, per point^§^ | 1.06 | 1.00-1.13 | 0.069 |
| Corticosteroids | 0.59 | 0.30-1.17 | 0.132 |
| Tocilizumab | 0.69 | 0.34-1.38 | 0.294 |
| Remdesivir | 0.76 | 0.27-2.14 | 0.603 |
| Prone positioning^§^ | 2.32 | 1.19-4.52 | 0.013 |
| Inhaled Nitric Oxide^§^ | 2.34 | 1.03-5.33 | 0.043 |
| PaO_2_/FiO_2_ ratio, per unit | 1.00 | 0.99-1.00 | 0.192 |

PEEP: Positive End-Expiratory Pressure; P_plat_: Plateau Pressure; PBW: Predicted Body weight; ^§^Variable considered for multivariable analysis.

**Table 3: Rates of Missing Data For Each Variable**

| Variable | N Missing (%) |
| --- | --- |
| Anesthesia machine | 0 (0) |
| Age at admission | 0 (0) |
| Sex | 0 (0) |
| Body mass index | 3 (3.4) |
| Race | 0 (0) |
| APACHE II Score | 4 (4.5) |
| SOFA Score | 28 (31.5) |
| Hypertension | 0 (0) |
| Diabetes mellitus | 1 (1.1) |
| Obesity | 1 (1.1) |
| COPD | 0 (0) |
| Hypercholesterolemia | 0 (0) |
| **Clinical variables** |  |
| PaO_2_/FiO_2_, mmHg | 5 (5.6) |
| PEEP, cmH_2_O | 3 (3.4) |
| P_plat_, cmH_2_O | 16 (18.0) |
| Vt/PBW (ml/kg) | 5 (5.6) |
| RR, breaths/min | 5 (5.6) |
| C_rs_, ml/cmH_2_O | 17 (19.1) |
| dP, cmH_2_O | 16 (18.0) |
| PaO_2_, mmHg | 5 (5.6) |
| PaCO_2_, mmHg | 5 (5.6) |
| pH | 8 (9.0) |
| HCO_3_^-^, mmol/l | 18 (20.2) |
| Base excess | 11 (12.4) |
| Lactate, mmol/l | 11 (12.4) |
| Heart rate, beats/min | 7 (7.9) |
| MAP, mmHg | 6 (6.7) |
| **Laboratory findings** |  |
| CRP, mg/dl | 14 (15.7) |
| Procalcitonin, ng/ml | 23 (25.8) |
| WBC, 10^9^/L | 5 (5.6) |
| Tot. lymphocytes, % | 12 (13.5) |
| Hematocrit, % | 6 (6.7) |
| Hemoglobin, mg/dl | 5 (5.6) |
| Platelets, 10^12^/L | 5 (5.6) |
| ALT, IU/L | 12 (13.5) |
| AST, IU/L | 17 (19.1) |
| LDH, IU/L | 18 (20.2) |
| Bilirubin, mg/dl | 17 (19.1) |
| Creatinine, mg/dl | 12 (13.5) |
| Glucose, mg/dl | 24 (27.0) |
| Sodium, mEq/l | 20 (22.5) |
| Potassium, mEq/l | 20 (22.5) |
| Creatine kinase, IU/l | 24 (27.0) |
| CK-MB, ng/ml | 42 (47.2) |
| Troponin-T, ng/l | 36 (40.4) |
| NT-proBNP, ng/l | 42 (47.2) |
| PT, s | 23 (25.8) |
| aPTT, s | 23 (25.8) |
| D-dimer, mcg/ml | 44 (39%) |
| **Treatments received, no. (%)** |  |
| Lopinavir/ritonavir | 0 (0) |
| Hydroxychloroquine | 0 (0) |
| Antibiotic prophylaxis | 0 (0) |
| Corticosteroids | 1 (1.1) |
| Tocilizumab | 1 (1.1) |
| Remdesivir | 1 (1.1) |
| Continuous IV sedation | 0 (0) |
| Continuous IV opioids | 0 (0) |
| Inhaled sedation | 0 (0) |
| Paralysis | 0 (0) |
| Prone positioning | 1 (1.1) |
| Inhaled Nitric Oxide | 1 (1.1) |
| **Outcomes** |  |
| Mortality | 0 (0) |
| ICU length of stay | 0 (0) |
| ICU-free days | 0 (0) |
| Hospital length of stay | 0 (0) |
| Hospital-free days | 0 (0) |
| Mechanical ventilation days | 0 (0) |
| Ventilator-free days | 0 (0) |
| ECMO initiation | 0 (0) |
| Tracheostomy | 0 (0) |
| Barotrauma | 0 (0) |
| Emergency tube exchange | 0 (0) |

*Abbreviations: APACHE II: Acute Physiology and Chronic Health Evaluation II; SOFA: Sequential Organ Failure Assessment; COPD: Chronic Obstructive Pulmonary Disease; PEEP: Positive End-Expiratory Pressure; P_plat_: Plateau Pressure; Vt: Tidal Volume; PBW: Predicted Body Weight; RR: Respiratory Rate; C_rs_: Respiratory System Compliance; dP: Driving Pressure (P_plat_ – PEEP); MAP: Mean Arterial Pressure; CRP: C-Reactive Protein; WBC: White Blood Cells; ALT: Alanine Aminotransferase; AST: Aspartate Aminotransferase; LDH: Lactate Dehydrogenase; NT-proBNP: N-Terminal pro B-type Natriuretic Peptide; PT: Prothrombin time; aPTT (activated partial thromboplastin time); IL-6: Interleukin-6; IV: Intravenous; ECMO: Extra-Corporeal Membrane Oxygenation; ICU: Intensive Care Unit.*

**Table 4: Frequency of Variable Inclusion in 100 Bootstrapped Samples**

| Variable | N Models Variable Included In |
| --- | --- |
| Anesthesia Machine*^§^* | 85 (85%) |
| Creatinine*^§^* | 89 (89%) |
| Hypertension*^§^* | 71 (71%) |
| Bilirubin*^§^* | 68 (68%) |
| Inhaled Nitric oxide | 63 (63%) |
| Hypercolesterolemia | 55 (55%) |
| Age | 55 (55%) |
| Diabetes mellitus | 51 (51%) |
| COPD | 50 (50%) |
| pH | 49 (49%) |
| Driving Pressure | 46 (46%) |
| Mean arterial blood pressure | 45 (45%) |
| Hematocrit | 44 (44%) |
| Hemoglobin | 44 (44%) |
| Lactate | 41 (41%) |
| *^§^Variable selected for final multivariable sensitivity analysis; Abbreviations: COPD = chronic obstructive pulmonary disorder* | |

**Table 5: Final Sensitivity Model Using Bootstrapped Samples**

| Variable | Hazard Ratio | 95% Confidence Interval | P value |
| --- | --- | --- | --- |
| Allocation to an Anesthesia Machine | 3.46 | 1.57-7.63 | 0.002 |
| Creatinine, per mg/dl | 8.37 | 3.13-22.39 | <0.001 |
| Hypertension | 3.35 | 1.46-7.69 | 0.005 |
| Bilirubin, per mg/dl | 1.52 | 1.20-1.94 | 0.001 |

**Day 1 Day 2 Day 7**

**Figure 1: Change in mean arterial pressure in the two groups (AM and ICU-VENT)**

*Data are presented at baseline, 48 hours and on day 7. The dots represent median value for MAP and the bar extremities represent the interquartile range. Differences between groups were assessed with a Mann-Whitney U test. * p=0.050*
